# Significant liver fibrosis is a predictor of poor health-related quality of life in people living with HIV

**DOI:** 10.1007/s11136-022-03232-w

**Published:** 2022-08-22

**Authors:** Maurice Michel, Alisha Wahl, Malena Anders, Saleh A. Alqahtani, Wolfgang M. Kremer, Peter R. Galle, Christian Labenz, Daniel Grimm, Martin Sprinzl, Jörn M. Schattenberg

**Affiliations:** 1 Metabolic Liver Research Program, I. Department of Medicine, University Medical Center of the Johannes Gutenberg-University, Mainz, Germany; 2 I. Department of Medicine, University Medical Center of the Johannes Gutenberg-University, Mainz, Germany; 3 Liver Transplant Center, King Faisal Specialist Hospital & Research Center, Riyadh, Saudi Arabia; 4 Division of Gastroenterology and Hepatology, Johns Hopkins University, Baltimore, MD USA

**Keywords:** HIV, HRQL, MOS-HIV, Significant fibrosis, VCTE, Metabolic comorbidities

## Abstract

**Purpose:**

Liver-related comorbidities can impair the health-related quality of life (HRQL) in people living with human immunodeficiency virus (HIV) (PLWH). However, the role of hepatic steatosis and significant fibrosis in PLWH remains incompletely characterized. Therefore, the aim of this study was to explore the association of hepatic steatosis and significant fibrosis on the HRQL using the medical outcomes study HIV health survey (MOS-HIV) in PLWH.

**Methods:**

A total of 222 PLWH were included in the final analysis of this cohort study. Metabolic comorbidities, socioeconomic factors, and HIV-related parameters were assessed. Hepatic steatosis and fibrosis were measured using vibration-controlled transient elastography (VCTE). The MOS-HIV survey, containing two summary scores (physical health summary (PHS) and mental health summary (MHS)) and ten domains, was used to assess the HRQL. Clinical predictors were identified using multivariable linear regression models.

**Results:**

The majority of this cohort was male, and the median age was 52 years, with a high prevalence of hepatic steatosis (*n* = 81, 36.5%). Significant fibrosis was present in 7.7% (*n* = 17). The mean PHS and MHS scores were 52.7 ± 9.5 and 51.4 ± 10.5, respectively. The lowest scores were in the general health perception (GHP) and energy/fatigue (EF) domains. A high BMI and waist circumference were associated with a poor PHS score. Lower education, unemployment, arterial hypertension, and significant fibrosis remained independent predictors of an impaired HRQL.

**Conclusion:**

Metabolic comorbidities, significant fibrosis, and a lower socioeconomic status may negatively affect the HRQL in PLWH. Considering the negative impact of significant fibrosis on the outcome, counseling and preventive measures according to current guidelines are recommended in this subgroup of PLWH.

**Supplementary Information:**

The online version contains supplementary material available at 10.1007/s11136-022-03232-w.

## Introduction

The life expectancy of people living with human immunodeficiency virus (HIV) (PLWH) has gradually been increasing over the last several decades as a result of advances in antiretroviral therapy (ART) [1]. HIV is nowadays considered a chronic disease requiring lifelong treatment. Although with significant improvements in HIV-related mortality and morbidity, physical and psychological well-being may be significantly impaired. Patients’ perceived and reported health aspects beyond objectively quantified clinical parameters are summarized as health-related quality of life (HRQL). PLWH consistently report lower HRQL than HIV-negative individuals despite antiretroviral therapy and viral suppression [2, 3]. Albeit the role of HIV-associated complications and side effects of ART on HRQL, psychosocial factors related to stigma, socioeconomic status, and limited access to social support may be other key determinants of HRQL [4–6]. In an aging HIV population, commonly seen age-related and metabolic comorbidities may further add to the burden on the HRQL [7].

Metabolic syndrome and its risk factors have been increasing and show a higher prevalence in PLWH compared to the general population [8]. Treatment with ART and HIV infection is known to be pro-steatogenic with worse metabolic outcomes—dependent on the chosen ART regimen [9, 10]. Nevertheless, an increasingly sedentary lifestyle with poor dietary and physical habits and a higher age impose an additional threat to the metabolic health of PLWH [11]. These factors have also led to a rise in the prevalence of hepatic steatosis, negatively impacting the HRQL in PLWH [12, 13]. In the absence of high alcohol intake and other secondary causes, hepatic steatosis is commonly referred to as non-alcoholic fatty liver disease (NAFLD). In this context, significant fibrosis is deemed a key driver and mediator of disease progression, liver-related complications, and mortality [14]. In addition, chronic liver diseases have been associated with adverse effects on the HRQL [15–17]. While metabolic risk factors and NAFLD negatively affect mental and physical health in HIV-negative individuals, little is known about their impact on the HRQL in PLWH [18, 19].

The HRQL can be assessed with either generic (i.e., the EQ-5D-5L) or disease-specific questionnaires [20, 21]. The medical outcomes study HIV health survey (MOS-HIV) is a disease-specific measure of HRQL and has been validated for use in PLWH and recommended as a suitable measure of HRQL in PLWH [22, 23]. The MOS-HIV contains ten domains that include HRQL-related aspects affected by liver diseases and other comorbidities [22]. Although MOS-HIV has been validated and shown to be impaired in PLWH and liver disease, little focus has been given to the impact of hepatic steatosis and significant fibrosis yet [24, 25].

Currently, only little data is available on hepatic steatosis and fibrosis and their role in affecting the HRQL in PLWH. Therefore, the aim of this study was to explore the association of hepatic steatosis and fibrosis on the HRQL in PLWH using the MOS-HIV survey.

## Materials and methods

### Inclusion and exclusion criteria

**Fig. 1:**
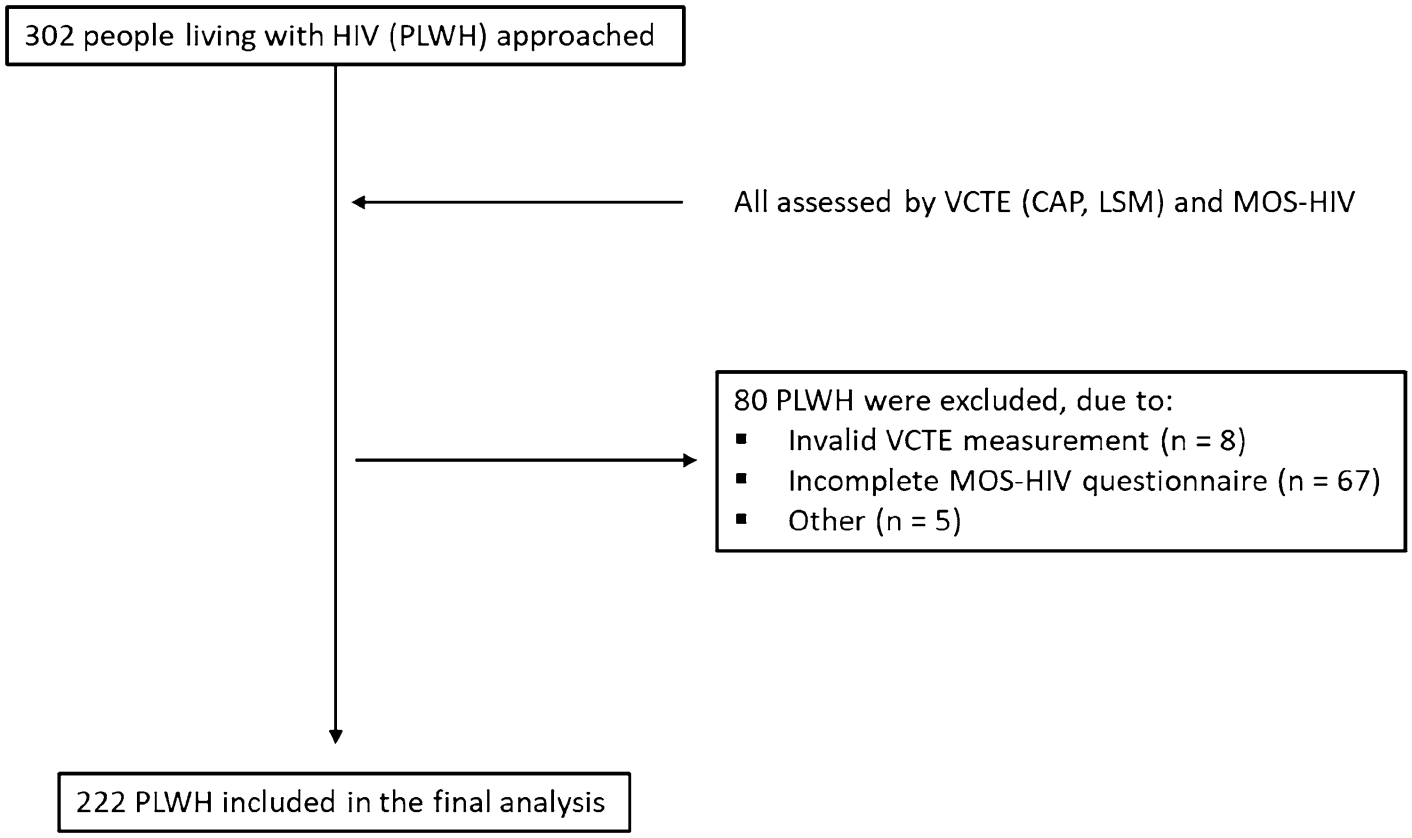
Flow diagram showing inclusion and exclusion criteria of study participants. VCTE vibration-controlled transient elastography, CAP controlled attenuation parameter, LSM liver stiffness measurement, MOS-HIV the medical outcomes study HIV health survey

Participants had to be at least 18 years of age, provide written informed consent, and have an HIV infection. PLWH with an active malignancy were excluded. Laboratory values and medical history were available from the electronic health care records. A total of 302 people living with HIV (PLWH) were approached and assessed by VCTE and the MOS-HIV survey between 2018 and 2021 in this cohort study (FLASH, Prevalence of Advanced Fibrosis in Patients Living With HIV, NCT04066608) at the outpatient clinic of the Metabolic Liver Research Program at the University Medical Center Mainz in Germany. 80 PLWH were excluded from the final analysis due to; (a) invalid VCTE measurements (*n* = 8), (b) an incomplete MOS-HIV questionnaire (*n* = 67) and (c) multiple other reasons (*n* = 5). A total of 222 PLWH meeting the inclusion criteria were used for this analysis (Fig. 1).

### Assessment of hepatic steatosis and significant fibrosis

Vibration-controlled transient elastography (VCTE, FibroScan® 430 mini; SMART Exam was introduced starting in 2020; Echosens, Paris, France) was used to assess hepatic steatosis and significant fibrosis by measuring the controlled attenuation parameter (CAP, dB/m) and liver stiffness measurement (LSM, kPa), respectively [26]. A CAP of ≥ 275 dB/m was chosen to define hepatic steatosis, according to current practice guidelines [27]. The M probe was used in *n* = 201 (90.5%), whereas the XL probe was used in *n* = 21 (9.5%) exams whenever suggested by the device. A total of eight participants were excluded due to invalid measurements. Significant fibrosis (≥ F2) was defined as an LSM ≥ 8.2 kPa [28]. Only measurements with an interquartile range (IQR) < 30% and a success rate > 70% were considered valid [29]. Non-alcoholic fatty liver disease was defined according to current practice guidelines and if alcohol intake was below 20 g/day (males) and 10 g/day (females) [30].

### Definition of clinical characteristics

Several characteristics, including sociodemographic variables, metabolic comorbidities, laboratory values, and HIV-related parameters, have been analyzed. Higher education was defined if participants had at least a high school degree, whereas a lower education implies that participants had no high school degree or above. The metabolic syndrome was defined according to the International Diabetes Federation (IDF) [31]. The definition of the metabolic syndrome requires evidence of central obesity (waist circumference in males ≥ 94 cm and females ≥ 80 cm) and at least two of the following four factors: a previously diagnosed type 2 diabetes, raised triglycerides (≥ 150 mg/dL, or treatment of this condition), low HDL cholesterol (males < 40 mg/dL; females < 50 mg/dL) and arterial hypertension (systolic ≥ 130 or diastolic ≥ 85 mmHg) [31]. Body mass index (BMI) was calculated using height and weight (BMI = weight [kg]/height^2^ [m^2^]). Alcohol intake was clinically assessed, and consumption above 20 g/day and 10 g/day for males and females was considered a higher alcohol intake. HIV RNA viral load was divided into two groups: below and above the threshold (50 copies/ml).

### The medical outcomes study HIV health survey (MOS-HIV)

For the assessment of the HRQL, the validated German version of the HIV-specific medical outcomes study HIV health survey (MOS-HIV) was used [22, 32]. The questionnaire has also been validated in PLWH and liver disease [24]. Overall, the MOS-HIV consists of the following ten domains that include 35 questions in total: general health perception (GHP), physical functioning (PF), role functioning (RF), social functioning (SF), cognitive functioning (CF), pain (P), mental health (MH), energy/fatigue (EF), health distress (HD) and quality of life (QL). Two summary scores, physical health summary (PHS) and mental health summary (MHS), are derived from the scores of the ten domains. Each score of the ten domains ranges from 0 to 100, with higher scores representing a better HRQL. The scores of PHS and MHS are transformed into t-scores with a mean of 50 and a standard deviation of 10.

### Ethics

All participants provided written informed consent. The study was conducted according to the ethical guidelines of the 1975 Declaration of Helsinki (6th revision, 2008). The ethics committee approved the study protocol of the Landesärztekammer Rhineland-Palatine (Nr. 873.199.10 (7208)).

### Statistical analysis

**Fig. 2:**
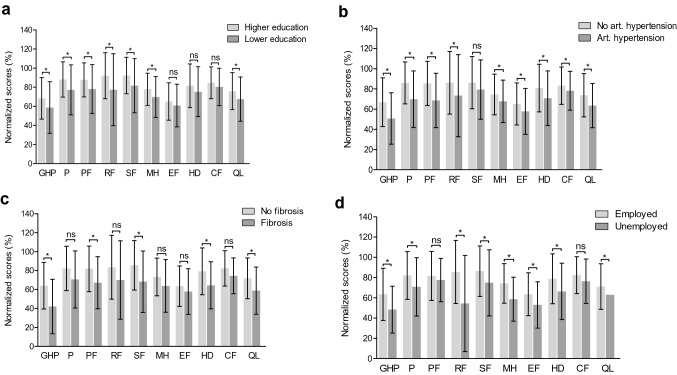
Comparison of each MOS-HIV domain concerning the predictors of higher and lower PHS and MHS scores in PLWH. **a** Higher education vs. lower education. **b** No arterial hypertension vs. arterial hypertension. **c** No fibrosis vs. fibrosis (LSM > 8.2 kPa). **d** Employed vs. unemployed. Mean values with standard deviation are shown. A higher value (%) indicates a better HRQL in the respective domain. A *p*-value < 0.05 was considered statistically significant (*p < 0.05). GHP general health perceptions, P pain, PF physical functioning, RF role functioning, SF social functioning, MH mental health, EF energy/fatigue, HD health distress, CF cognitive functioning, QL quality of life

Quantitative data are presented as median values with interquartile ranges (IQR 25th; 75th) or mean values with standard deviation (± SD). Categorical variables are shown as frequencies with percentages. The Mann–Whitney U rank test was used to compare differences between groups of continuous variables (as seen in Fig. 2a–d and Supplementary Tables 1–5). The chi-squared test was applied to compare two or more patient groups with categorical variables. All tests were two-tailed, and statistically significant values were defined as *p* < 0.05. Univariable analysis was used to examine associations between demographic and clinical variables with the PHS and MHS of the MOS-HIV. All variables with *p* < 0.05 in the univariable analysis and the two clinical parameters age and sex were then analyzed in a multivariable linear regression model. IBM SPSS Statistic Version 23.0 (Armonk, NY: IBM Corp.) was used for all data analysis and statistical tests. GraphPad Prism 5.0 (San Diego, CA: GraphPad Software, LLC) was used for all graphs.

## Results

### Baseline characteristics and demographics of PLWH

A total of 222 PLWH were included for the final analysis in this study. The median age was 52 years (IQR 42; 58), and the median time since diagnosis was 12 years (IQR 7; 21). The majority of PLWH were male sex (70.7%). Unemployment was noticed in 12.2% (*n* = 27) and a lower education was seen in 64.9% (*n* = 144). The median CAP was 245.5 dB/m (IQR 213.8; 300.3). A total of 81 (36.5%) PLWH had hepatic steatosis, of whom 70 (31.5%) participants fulfilled the criteria of a NAFLD. The median LSM was 4.6 kPa (IQR 3.9; 5.7), and significant fibrosis was present in 7.7% (*n* = 17). Arterial hypertension was present in 28.4%. The median BMI and waist circumference were 25.2 kg/m^2^ and 97.0 cm, respectively. CDC stage C was present in 20.3% (*n* = 45) of PLWH. Most PLWH were treated with TAF-containing regimens (*n* = 143, 64.4%) and 59.9% (*n* = 133) also received an INSTI as part of the ART regimen. These and other general characteristics of the cohort are summarized in Table 1.

**Table 1:** Baseline characteristics and demographics of PLWH

Variable	Total cohort (*n* = 222)
	*n* (% or IQR)
General characteristics	
Age in years	52.0 (42.0; 58.0)
Time since diagnosis (years) *n* = 211	12.0 (7.0; 21.0)
Sex, male/female	157 (70.7)/65 (29.3)
Unemployed *n* = 208	27 (12.2)
Lower education *n* = 208	144 (64.9)
VCTE	
CAP (dB/m)	245.5 (213.8; 300.3)
Hepatic steatosis ≥ 275 dB/m	81 (36.5)
LSM (kPa)	4.6 (3.9; 5.7)
Significant fibrosis ≥ 8.2 kPa	17 (7.7)
NAFLD	70 (31.5)
Metabolic comorbidities	
Hyperlipidemia	58 (26.1)
Arterial hypertension	63 (28.4)
Type 2 diabetes	22 (9.9)
Metabolic syndrome	23 (10.4)
BMI (kg/m^2^) *n* = 218	25.2 (22.4; 28.7)
Waist circumference (cm) *n* = 217	97.0 (87.0; 104.0)
High alcohol intake	19 (8.6)
Hypothyroidism *n* = 187	12 (5.4)
Sleep apnea syndrome *n* = 186	43 (19.4)
Laboratory values	
ALT (U/l) *n* = 210	23.5 (18.0; 33.0)
AST (U/l) *n* = 210	26.0 (23.0; 32.0)
Albumin *n* = 124	40.0 (38.0; 42.0)
Triglycerides (mg/dL) *n* = 139	136.0 (94.0; 195.0)
Cholesterol (mg/dL) *n* = 141	201.0 (175.5; 231.0)
Leukocytes (/nl) *n* = 218	6.4 (5.3; 7.8)
Hemoglobin (g/dL) *n* = 218	14.9 (13.7; 15.7)
HIV-related parameters	
CD4 + (cells/µl) n = 212	739.0 (524.5; 941.5)
HIV RNA viral load n = 216	
Below threshold	154 (69.4)
Above threshold	62 (27.9)
CDC stage C	45 (20.3)
NRTI	200 (90.1)
TAF/TDF as part of ART	143 (64.4)/25 (11.3)
INSTI	133 (59.9)

Data are expressed as numbers, median, percentage (%), or interquartile ranges (IQR 25th; 75th)

*ALT* alanine-aminotransaminase, *AST* aspartate-aminotransaminase, *BMI* body mass index, *CDC* centers for disease control and prevention, *INSTI* integrase inhibitors, *TAF* tenofovir alafenamide, *TDF* tenofovir disoproxil fumarate, *NAFLD* non-alcoholic fatty liver disease, *NRTI* nucleoside reverse-transcriptase inhibitors

### The MOS-HIV in PLWH

The median physical health summary (PHS) and mental health summary (MHS) scores were 56.1 (IQR 48.7; 60.0) and 53.7 (IQR 44.9; 59.5), respectively. The highest scores were seen in the domains physical functioning (PF), role functioning (RF), social functioning (SF), and pain (P). In turn, the lowest scores were seen in the domains of general health perception (GHP), energy/fatigue (EF), and quality of life (QL). The median and mean values of the MOS-HIV are summarized in Table 2.

**Table 2:** Median and mean values of the MOS-HIV survey

Variable	Total cohort (*n* = 222)	
MOS-HIV	Median (25th; 75th)	Mean (± SD)
Summary scores		
Physical health summary (PHS) *n* = 207	56.1 (48.7; 60.0)	52.7 ± 9.5
Mental health summary (MHS) *n* = 207	53.7 (44.9; 59.5)	51.4 ± 10.5
Dimension scores		
General health perception (GHP) *n* = 217	70.0 (45.0; 80.0)	62.3 ± 25.5
Physical functioning (PF) *n* = 220	91.7 (75.0; 100.0)	80.8 ± 24.5
Role functioning (RF) *n* = 215	100.0 (100.0; 100.0)	82.6 ± 24.5
Social functioning (SF) *n* = 222	100.0 (80.0; 100.0)	84.2 ± 27.0
Cognitive functioning (CF) *n* = 219	85.0 (75.0; 100.0)	81.8 ± 18.8
Pain (P) *n* = 222	94.4 (66.6; 100.0)	81.4 ± 24.1
Mental health (MH) *n* = 220	76.0 (60.0; 88.0)	72.5 ± 20.6
Energy/fatigue (EF) *n* = 219	65.0 (50.0; 80.0)	63.1 ± 21.6
Health distress (HD) *n* = 219	85.0 (60.0; 100.0)	78.1 ± 24.9
Quality of life (QL) *n* = 221	75.0 (50.0; 75.0)	70.8 ± 22.0

Data are expressed as median values with interquartile range (IQR 25th; 75th) or means with standard deviation (± SD)

### Clinical predictors for a poor PHS

In the univariable analysis, a longer disease duration (time since diagnosis) (β − 0.164, 95% CI − 0.302, − 0.025), female sex (β − 0.141, 95% CI − 0.284, − 0.005), a lower education (β − 0.234, 95% CI − 0.369, − 0.095), unemployment (β − 0.171, 95% CI − 0.337, − 0.034), a diagnosis of sleep apnea syndrome (β − 0.168, 95% CI − 0.315, − 0.021), a high BMI (β − 0.158, 95% CI − 0.295, − 0.021), a high waist circumference (β − 0.231, 95% CI − 0.360, − 0.093), arterial hypertension (β − 0.345, 95% CI − 0.476, − 0.217) and significant fibrosis (β − 0.176, 95% CI − 0.330, − 0.043) were associated with an impaired PHS. In the multivariable analysis, a lower education (β − 0.148, 95% CI − 0.295, − 0.002), arterial hypertension (β − 0.303, 95% CI − 0.487, − 0.145) and significant fibrosis (β − 0.214, 95% CI − 0.377, − 0.071) remained the only independent predictors of a poor PHS (Table 3).

**Table 3:** Uni- and multivariable analyses of clinical and laboratory parameters with the summary score PHS

Variable	PHS					
	Univariable analysis			Multivariable analysis^**†**^		
	*β*	95% CI	*p*	*β*	95% CI	*p*
Age	− 0.106	− 0.239, 0.030	0.128	0.030	− 0.183, 0153	0.858
Time since diagnosis	− **0.164**	− **0.302, **− **0.025**	**0.021**	− 0.070	− 0.238, 0.094	0.392
Sex, female	− **0.141**	− **0.284, **− **0.005**	**0.042**	− 0.103	− 0.262, 0.050	0.182
Lower education	− **0.234**	− **0.369, **− **0.095**	**0.001**	− **0.148**	− **0.295, **− **0.002**	**0.046**
Unemployment	− **0.171**	− **0.337, **− **0.034**	**0.017**	− 0.120	− 0.289, 0.024	0.097
Hypothyroidism	0.018	− 0.126, 0.162	0.807			
Sleep apnea syndrome	− **0.168**	− **0.315, **− **0.021**	**0.026**	− 0.038	− 0.185, 0.108	0.605
BMI	− **0.158**	− **0.295, **− **0.021**	**0.024**	0.030	− 0.121, 0.197	0.705
Waist circumference	− **0.231**	− **0.360, -0.093**	**0.001**			
High alcohol intake	− 0.038	− 0.174, 0.099	0.590			
Type 2 diabetes	0.014	− 0.128, 0.156	0.847			
Arterial hypertension	− **0.345**	− **0.476, **− **0.217**	** < 0.001**	− **0.303**	− **0.487, **− **0.145**	** < 0.001**
Hyperlipidemia	− 0.005	− 0.147, 0.136	0.938			
Metabolic syndrome	− 0.022	− 0.158, 0.114	0.754			
Hepatic steatosis	− 0.049	− 0.187, 0.088	0.479			
Significant fibrosis	− **0.176**	− **0.330, **− **0.043**	**0.011**	**− 0.214**	**− 0.377, − 0.071**	**0.004**
ALT (U/l)	0.029	− 0.106, 0.161	0.686			
AST (U/l)	− 0.044	− 0.177, 0.093	0.541			
Triglycerides	0.026	− 0.152, 0.205	0.769			
Cholesterol	0.042	− 0.142, 0.232	0.633			
Leukocytes	− 0.030	− 0.167, 0.108	0.671			
HIV RNA	− 0.061	− 0.197, 0.077	0.388			
CD4 cells/µl	0.030	− 0.111, 0.171	0.674			
CDC stage C	0.041	− 0.121, 0.202	0.620			
TAF vs. TDF	0.038	− 0.127, 0.208	0.635			
INSTI	− 0.101	− 0.246, 0.043	0.167			

Univariable and multivariable linear regression analysis was done. With all factors showing a *p*-value < 0.05 and the clinical parameters age and sex, a multivariable linear regression model was built

*CI* confidence interval and *β* beta show each standardized values

Boldface indicates statistical significance. A *p*-value < 0.05 was considered statistically significant

^**†**^Multivariable linear regression analysis (*n* = 166): age, sex, time since diagnosis, lower education, unemployment, sleep apnea syndrome, BMI, arterial hypertension, and significant fibrosis. Waist circumference was excluded from the analysis to avoid multicollinearity with the variable BMI

### Clinical predictors for a poor MHS

A lower education (β − 0.163, 95% CI − 0.303, − 0.023), unemployment (β − 0.184, 95% CI − 0.354, − 0.049), arterial hypertension (β − 0.221, 95% CI − 0.357, − 0.087) and significant fibrosis (β − 0.165, 95% CI − 0.318, − 0.031) were associated with an impaired MHS in the univariable analysis. In the multivariable analysis, unemployment (β − 0.164, 95% CI − 0.329, − 0.030), arterial hypertension (β − 0.187, 95% CI − 0.334, − 0.041) and significant fibrosis (β − 0.167, 95% CI − 0.316, − 0.032) were independent predictors of a poor MHS (Table 4).

**Table 4:** Uni- and multivariable analyses of clinical and laboratory parameters with the summary score MHS

Variable	MHS					
	Univariable analysis			Multivariable analysis^**†**^		
	*β*	95% CI	*p*	*β*	95% CI	*p*
Age	0.002	− 0.133, 0.138	0.975	0.064	− 0.185, 0.087	0.392
Time since diagnosis	− 0.025	− 0.168, 0.117	0.728			
Sex, female	− 0.114	− 0.257, 0.023	0.102	− 0.071	− 0.218, 0.072	0.321
Lower education	− **0.163**	− **0.303, **− **0.023**	**0.023**	− 0.107	− 0.248, 0.034	0.137
Unemployment	− **0.184**	− **0.354, **− **0.049**	**0.010**	− **0.164**	− **0.329, **− **0.030**	**0.019**
Hypothyroidism	0.034	− 0.110, 0.176	0.652			
Sleep apnea syndrome	− 0.130	− 0.273, 0.018	0.085			
BMI	− 0.069	− 0.207, 0.069	0.327			
Waist circumference	− 0.112	− 0.246, − 0.026	0.112			
High alcohol intake	0.010	− 0.127, 0.146	0.888			
Type 2 diabetes	− 0.008	− 0.151, 0.134	0.911			
Arterial hypertension	− **0.221**	− **0.357, **− **0.087**	**0.001**	− **0.187**	− **0.334, **− **0.041**	**0.012**
Hyperlipidemia	0.093	− 0.045, 0.237	0.181			
Metabolic syndrome	0.047	− 0.089, 0.183	0.499			
Hepatic steatosis	0.017	− 0.120, 0.155	0.806			
Significant fibrosis	− **0.165**	− **0.318, **− **0.031**	**0.018**	− **0.167**	− **0.316, **− **0.032**	**0.017**
ALT (U/l)	− 0.039	− 0.169, 0.097	0.590			
AST (U/l)	− 0.099	− 0.229, 0.040	0.168			
Triglycerides	0.095	− 0.080, 0.274	0.281			
Cholesterol	0.079	− 0.100, 0.271	0.365			
Leukocytes	0.052	− 0.086, 0.190	0.458			
HIV RNA	− 0.067	− 0.204, 0.072	0.348			
CD4 cells/µl	0.037	− 0.106, 0.181	0.610			
TAF vs. TDF	0.064	− 0.099, 0.235	0.421			
INSTI	− 0.111	− 0.257, − 0.033	0.128			

Univariable and multivariable linear regression analysis was done. With all factors showing a *p*-value < 0.05 and the clinical parameters age and sex, a multivariable linear regression model was built

*CI* confidence interval and *β* beta show each standardized values

Boldface indicates statistical significance. A *p*-value < 0.05 was considered statistically significant

^**†**^Multivariable linear regression analysis (*n* = 196): age, sex, lower education, unemployment, arterial hypertension, and significant fibrosis

### The MOS-HIV domain scores concerning the predictors of an impaired HRQL in PLWH

In the next step, we compared each summary and domain score of the MOS-HIV within the previously identified predictors of a poor HRQL (Fig. 2a–d, Supplementary Tables 1–4). Individuals with lower education had overall worse PHS and MHS scores compared with higher education (Supplementary Table 1). The GHP, PF, RF, SF, P, MH, and QL domains were significantly lower in PLWH and lower education. The highest scores were seen in the RF and SF domains. No difference was seen in the CF, EF, and HD domains (Fig. 2a). The two summary scores were lower in PLWH and arterial hypertension (Supplementary Table 2). Similar aspects were seen in all other domains except the SF domain (Fig. 2b). PHS and MHS summary scores were significantly lower in PLWH and significant fibrosis (Supplementary Table 3), and they were numerically the lowest compared with the other negative predictors (lower education, arterial hypertension, and unemployment). In addition, the GHP and QL domains showed the lowest scores in this subgroup. In contrast, no difference was seen in the RF, CF, P, MH, and EF domains (Fig. 2c). Unemployment had a lower score in the MHS summary score, and no difference was seen in the PHS (Supplementary Table 4). In this subgroup, the lowest scores were detected in the GHP, RF, and EF domains (Fig. 2d). The complete statistical analysis is provided in Supplementary Tables 1–4. In line with the univariable analyses, the scores of the PHS and MHS and the domains of the MOS-HIV, except the cognitive function domain, showed no difference in PLWH and hepatic steatosis (Supplementary Table 5).

## Discussion

In this study, we analyzed the association of hepatic steatosis and fibrosis on the HRQL in PLWH using the MOS-HIV. Although the MOS-HIV is a specific measure of HIV infection, it captures several aspects that are also relevant in assessing patients with liver disease. It combines two summary scores that assess physical (PHS) and mental health (MHS) based on ten domain scores, each evaluating various aspects of someone’s HRQL. In this context, general health perception (GHP) and energy/fatigue (EF) were two of the most burdensome HRQL aspects in this cohort. Arterial hypertension remained an independent predictor of impaired PHS and MHS scores. Lower education and unemployment were independently associated with poor PHS and MHS scores, respectively. To assess the effect of liver disease on the HRQL in PLWH, we included hepatic steatosis and significant fibrosis, measured non-invasively by VCTE, in our analysis. Significant fibrosis remained an independent predictor of a poor HRQL in both summary scores (PHS, MHS), and the GHP domain was the most burdensome in this subgroup. Overall, the lowest PHS and MHS scores were seen in individuals with significant fibrosis.

Despite the impact of hepatic steatosis and fibrosis on the HRQL in HIV-negative individuals, the association in PLWH remains largely unknown. Hepatic steatosis can be an initiating event for more severe liver injury due to inflammation (steatohepatitis) that promotes scarring of liver tissue (fibrosis). Significant fibrosis can lead to severe liver-related complications and higher mortality [14]. In patients with biopsy-confirmed NASH, inflammation and fibrosis were associated with a lower HRQL [17]. In this study, significant fibrosis remained an independent predictor of poor physical and mental health (PHS and MHS). These findings were also reflected in lower scores on several domains of the MOS-HIV compared to individuals without fibrosis. Although Henderson et al. analyzed the HRQL in PLWH and liver disease, most patients had hepatitis B and C coinfection, and information on liver fibrosis was not assessed [24]. Contrary to our previous analysis using a generic HRQL questionnaire (EQ-5D-5L), hepatic steatosis was not associated with a worse HRQL using the MOS-HIV survey in this study [13]. The metabolic risk profile was more prominent in the subgroup with significant fibrosis. Obesity (≥ 30 kg/m^2^) and a high waist circumference may result from poor dietary habits and a lack of physical activity. A lower physical health impacts mental health since the lack of physical activity often aggravates mental well-being in PLWH [33]. However, physical exercise may not be amenable for all of these patients due to a high BMI and limited adherence to exercise programs [34]. Sociodemographic factors may also influence these risk factors. Previous studies showed a lower HRQL in PLWH with low income [35]. A recent study revealed that lower education was associated with poorer metabolic health and a higher prevalence of NAFLD with significant fibrosis [36]. Here we show that a lower education remains an independent predictor of a poor PHS score. Moreover, the GHP domain showed the lowest scores in this subgroup, which implies a poor personal view of the own health. Overall, PLWH and significant fibrosis may characterize a population at risk that needs closer monitoring and counseling to improve the negative impact on HRQL.

Previous studies have often highlighted a higher prevalence of mental health problems, including anxiety and depression, in PLWH [37, 38]. In our study, the mental health domain had an overall lower score compared to other domains, although no difference was seen in the fibrosis subgroup. HIV infection and worries related to stigma and the chronic health condition may negatively impact mental health more than other comorbidities [39]. Unemployment remained an independent predictor of a lower MHS score. Interestingly, role functioning showed one of the lowest scores in the subgroup analysis. Additionally, PLWH with lower education had lower scores in the two domains, role- and social functioning. Sociodemographic factors have shown a high impact on the HRQL in PLWH, suggesting the need for more social support [2, 35]. Overall, social support may help reduce the effects of HIV-related stigmatization within the context of economic insecurity [4]. Thus, mental health may also be influenced by socioeconomic status with a significant impact on the HRQL in PLWH.

The energy/fatigue domain showed one of the lowest scores in this study. Fatigue is a common symptom, especially in patients with chronic liver diseases [15, 16]. In this study, fatigue was more burdensome in the fibrosis subgroup, although with no significant difference between these groups. Therefore, other factors may affect the EF domain more than significant fibrosis in PLWH alone. Although HIV-related parameters showed no association with a lower HRQL in this study, the use of INSTI correlated with the EF domain (data not shown). Common side effects of INSTI are sleep disturbances and depression in some cases [40]. Moreover, specific ART regimens are known to be pro-steatogenic [10]. Only recently, an impact of INSTI and TAF on weight gain and an increase in hepatic steatosis has been suggested in PLWH [9]. Thus, certain therapies for HIV may have opposite effects on metabolic outcomes and the HRQL, although longitudinal studies are needed to verify these results.

The median values of the two summary scores, PHS and MHS, in this study were comparable to an analysis from Ireland, although the median value of MHS was slightly lower [41]. Similar to our study, the cohort from Ireland included individuals with a well-controlled HIV infection. Despite improvements in the treatment of HIV and lower HIV-related mortality, the HRQL was still lower compared to the general population in the Irish cohort [41]. However, a comparison to the general population is lacking in our study due to missing data related to MOS-HIV from German HIV-negative individuals. Lower PHS and MHS values were also detected in a Canadian study and one study from China [42, 43]. The EF and GHP domains showed the lowest scores overall, which is in line with these recent studies [41, 42]. Overall, cultural and country-specific differences need to be considered in the assessment of HRQL [37].

### Strengths and limitations

This study has several limitations. The cross-sectional design limits the ability to assess causation, and longitudinal studies are needed to determine causality. To assess steatosis and significant fibrosis, we relied on non-invasive tests (NITs), although liver biopsy currently remains the reference standard to define hepatic fibrosis [44]. On the other hand, liver biopsy has several limitations and therefore is an unsuitable screening tool [45]. Furthermore, we included patients with mixed etiology of hepatic steatosis, including alcohol intake and secondary causes. However, most PLWH in this cohort fulfilled the criteria of a NAFLD. Although the MOS-HIV has also been validated for use in PLWH and liver disease, it is not a specific measure to assess liver-related HRQL aspects [24]. Liver-specific questionnaires may not cover all HIV infection and treatment aspects. Moreover, the MOS-HIV covers various domains relevant to other diseases, supporting its use as a generic measure in PLWH and other comorbid diseases [22]. The strength of this monocentric study is the large and well-characterized cohort of HIV-positive individuals. This study adds evidence on the effect of liver-related comorbidities on the HRQL in PLWH. Moreover, the MOS-HIV allowed identifying significant fibrosis as a negative predictor of physical and mental health. As outlined above, this study shows that significant fibrosis is not merely a clinical parameter but reflects an overall deprived population that requires social support and closer healthcare monitoring.

## Conclusion

This study identified several predictors of poor HRQL in PLWH. Moreover, PLWH and significant fibrosis may reflect a population at risk that need closer monitoring to improve liver-related and patient-reported outcomes. Targeted interventions and counseling in patients with hepatic steatosis and fibrosis could be an aspect of overcoming the impaired HRQL in PLWH.

## Supplementary Information

Below is the link to the electronic supplementary material.

Supplementary file1 (DOCX 29 kb)

## Abbreviations

ALT: Alanine transaminase
ART: Antiretroviral therapy
AST: Aspartate transaminase
BMI: Body mass index
CAP: Controlled attenuation parameter
GGT: Gamma-glutamyl transferase
INSTI: Integrase inhibitors
IQR: Interquartile range
LSM: Liver stiffness measurement
NAFLD: Non-alcoholic fatty liver disease
NASH: Non-alcoholic steatohepatitis
NIT: Non-invasive test
NRTI: Nucleoside reverse-transcriptase inhibitors
PLWH: People living with HIV
TAF: Tenofovir alafenamide
TDF: Tenofovir disoproxil fumarate
VCTE: Vibration-controlled transient elastography
