# Supplementary material for: Significant liver fibrosis is a predictor of poor health-related quality of life in people living with HIV
Source: Qual Life Res. 2022 Aug 22;32(2):401–11. doi: 10.1007/s11136-022-03232-w (PMC9911489; doi:10.1007/s11136-022-03232-w)
Supplement: Supplementary file 1 — Supplementary file1 (DOCX 29 kb) [file 11136_2022_3232_MOESM1_ESM.docx]

**Supplement**

**Supplementary Table 1 Comparison of the summary and domain scores in PLWH and higher education vs. lower education**

| **Variable** | **Higher education** | **Lower education** | **p-value** |
| --- | --- | --- | --- |
| **MOS-HIV** | **Mean (± SD)** | **Mean (± SD)** |  |
| **Summary scores** |  |  |  |
| Physical Health Summary n=207 | 55.8 ± 6.4 | 51.0 ± 10.3 | **0.002** |
| Mental Health Summary n=207 | 53.6 ± 8.8 | 49.9 ± 11.1 | **0.034** |
| **Domain scores** |  |  |  |
| General Health Perception n=217 | 68.3 ± 21.7 | 58.6 ± 27.0 | **0.017** |
| Physical Functioning n=220 | 87.6 ± 17.9 | 78.1 ± 25.6 | **0.011** |
| Role Functioning n=215 | 92.1 ± 24.1 | 77.3 ± 37.7 | **0.004** |
| Social Functioning n=222 | 92.2 ± 19.1 | 81.5 ± 28.3 | **0.008** |
| Cognitive Functioning n=219 | 84.5 ± 16.7 | 80.3 ± 19.5 | 0.156 |
| Pain n=222 | 88.1 ± 18.4 | 77.2 ± 26.1 | **0.009** |
| Mental Health n=220 | 77.8 ± 16.9 | 69.7 ± 21.4 | **0.012** |
| Energy/Fatigue n=219 | 65.0 ± 19.5 | 60.8 ± 22.4 | 0.315 |
| Health Distress n=219 | 81.4 ± 22.8 | 75.2 ± 26.2 | 0.185 |
| Quality of Life n=221 | 75.8 ± 19.4 | 67.5 ± 23.1 | **0.027** |

Data are expressed as mean values with standard deviation (± SD). A p-value < 0.05 was considered statistically significant.

**Supplementary Table 2 Comparison of the summary and domain scores in PLWH and no arterial hypertension vs. arterial hypertension**

| **Variable** | **No hypertension** | **Hypertension** | **p-value** |
| --- | --- | --- | --- |
| **MOS-HIV** | **Mean (± SD)** | **Mean (± SD)** |  |
| **Summary scores** |  |  |  |
| Physical Health Summary n=207 | 53.7 ± 8.3 | 47.5 ± 10.4 | **< 0.001** |
| Mental Health Summary n=207 | 52.9 ± 9.9 | 47.7 ± 11.0 | **0.001** |
| **Domain scores** |  |  |  |
| General Health Perception n=217 | 66.9 ± 24.1 | 50.7 ± 25.4 | **< 0.001** |
| Physical Functioning n=220 | 85.4 ± 21.8 | 68.6 ± 26.9 | **< 0.001** |
| Role Functioning n=215 | 86.1 ± 30.9 | 73.3 ± 40.6 | **0.017** |
| Social Functioning n=222 | 86.2 ± 25.8 | 79.9 ± 29.4 | 0.092 |
| Cognitive Functioning n=219 | 83.2 ± 18.5 | 78.2 ± 19.2 | **0.039** |
| Pain n=222 | 85.9 ± 20.8 | 69.8 ± 27.9 | **< 0.001** |
| Mental Health n=220 | 74.5 ± 20.2 | 67.7 ± 21.0 | **0.018** |
| Energy/Fatigue n=219 | 65.1 ± 20.8 | 57.8 ± 22.7 | **0.036** |
| Health Distress n=219 | 80.8 ± 23.6 | 70.8 ± 27.0 | **0.005** |
| Quality of Life n=221 | 73.7 ± 21.5 | 63.5 ± 21.9 | **0.001** |

Data are expressed as mean values with standard deviation (± SD). A p-value < 0.05 was considered statistically significant.

**Supplementary Table 3 Comparison of the summary and domain scores in PLWH and no fibrosis vs. fibrosis**

| **Variable** | **No fibrosis** | **Fibrosis** | **p-value** |
| --- | --- | --- | --- |
| **MOS-HIV** | **Mean (± SD)** | **Mean (± SD)** |  |
| **Summary scores** |  |  |  |
| Physical Health Summary n=207 | 53.2 ± 9.1 | 46.5 ± 12.2 | **0.022** |
| Mental Health Summary n=207 | 51.9 ± 10.2 | 45.0 ± 12.8 | **0.039** |
| **Domain scores** |  |  |  |
| General Health Perception n=217 | 63.9 ± 24.5 | 42.1 ± 28.8 | **0.002** |
| Physical Functioning n=220 | 81.8 ± 24.0 | 67.2 ± 27.5 | **0.013** |
| Role Functioning n=215 | 83.5 ± 33.6 | 70.0 ± 41.4 | 0.109 |
| Social Functioning n=222 | 85.6 ± 26.2 | 68.2 ± 32.4 | **0.011** |
| Cognitive Functioning n=219 | 82.4 ± 18.7 | 74.4 ± 18.9 | 0.060 |
| Pain n=222 | 82.3 ± 23.4 | 70.6 ± 30.2 | 0.136 |
| Mental Health n=220 | 73.2 ± 19.9 | 63.8 ± 27.8 | 0.283 |
| Energy/Fatigue n=219 | 63.5 ± 21.4 | 57.8 ± 24.1 | 0.331 |
| Health Distress n=219 | 79.1 ± 24.7 | 64.4 ± 24.8 | **0.012** |
| Quality of Life n=221 | 71.8 ± 21.5 | 58.8 ± 24.9 | **0.044** |

Data are expressed as mean values with standard deviation (± SD). A p-value < 0.05 was considered statistically significant.

**Supplementary Table 4 Comparison of the summary and domain scores in PLWH and employment vs. unemployment**

| **Variable** | **Employment** | **Unemployment** | **p-value** |
| --- | --- | --- | --- |
| **MOS-HIV** | **Mean (± SD)** | **Mean (± SD)** |  |
| **Summary scores** |  |  |  |
| Physical Health Summary n=207 | 53.1 ± 8.8 | 47.9 ± 12.7 | 0.078 |
| Mental Health Summary n=207 | 51.8 ± 10.3 | 45.5 ± 11.3 | **0.010** |
| **Domain scores** |  |  |  |
| General Health Perception n=217 | 63.4 ± 25.7 | 48.3 ± 23.1 | **0.004** |
| Physical Functioning n=220 | 81.6 ± 24.2 | 77.5 ± 21.4 | 0.202 |
| Role Functioning n=215 | 85.5 ± 31.1 | 54.3 ± 47.5 | **< 0.001** |
| Social Functioning n=222 | 86.3 ± 24.9 | 74.8 ± 32.6 | **0.050** |
| Cognitive Functioning n=219 | 82.4 ± 18.2 | 76.3 ± 21.8 | 0.168 |
| Pain n=222 | 82.0 ± 23.5 | 70.8 ± 28.8 | **0.021** |
| Mental Health n=220 | 74.2 ± 19.5 | 58.6 ± 21.7 | **< 0.001** |
| Energy/Fatigue n=219 | 63.5 ± 21.1 | 52.9 ± 22.8 | **0.023** |
| Health Distress n=219 | 78.8 ± 24.6 | 66.3 ± 27.8 | **0.017** |
| Quality of Life n=221 | 71.1 ± 22.5 | 62.9 ± 20.1 | **0.045** |

Data are expressed as mean values with standard deviation (± SD). A p-value < 0.05 was considered statistically significant.

**Supplementary Table 5 Comparison of the summary and domain scores in PLWH and no steatosis vs. steatosis**

| **Variable** | **No steatosis** | **Steatosis** | **p-value** |
| --- | --- | --- | --- |
| **MOS-HIV** | **Mean (± SD)** | **Mean (± SD)** |  |
| **Summary scores** |  |  |  |
| Physical Health Summary | 53.1 ± 9.2 | 52.1 ± 9.9 | 0.456 |
| Mental Health Summary | 51.3 ± 10.4 | 51.6 ± 10.8 | 0.596 |
| **Domains scores** |  |  |  |
| General Health Perception | 63.9 ± 24.8 | 59.2 ± 26.6 | 0.243 |
| Physical Functioning | 80.8 ± 24.9 | 80.8 ± 24.1 | 0.695 |
| Role Functioning | 82.8 ± 34.5 | 82.1 ± 34.2 | 0.744 |
| Social Functioning | 84.4 ± 27.8 | 83.9 ± 25.6 | 0.463 |
| Cognitive Functioning | 80.5 ± 17.9 | 84.0 ± 20.2 | **0.032** |
| Pain | 81.2 ± 23.8 | 81.6 ± 24.9 | 0.900 |
| Mental Health | 72.5 ± 20.5 | 72.5 ± 20.8 | 0.815 |
| Energy/Fatigue | 63.0 ± 21.6 | 63.2 ± 21.6 | 0.852 |
| Health Distress n=219 | 78.1 ± 24.5 | 77.9 ± 25.8 | 0.770 |
| Quality of Life n=221 | 70.7 ± 22.4 | 70.9 ± 21.5 | 0.975 |

Data are expressed as mean values with standard deviation (± SD). A p-value < 0.05 was considered statistically significant.
